# Direct and virtual measurements of abdominal aortic aneurysms: three-dimensional printed models

**DOI:** 10.1590/0100-3984.2019.0117

**Published:** 2021

**Authors:** Giovanna Ricarte Granja Gomes, Marcos Cordeiro D’Ornellas, Gustavo Nogara Dotto

**Affiliations:** 1 Universidade Federal de Santa Maria (UFSM), Santa Maria, RS, Brazil.

**Keywords:** Aortic aneurysm, abdominal/diagnostic imaging, Printing, three-dimensional, Aortic diseases, Biocompatible materials, Endovascular procedures, Blood vessel prosthesis implantation, Aneurisma da aorta abdominal/diagnóstico por imagem, Impressão tridimensional, Doenças da aorta, Materiais biocompatíveis, Procedimentos endovasculares, Implante de prótese vascular

## Abstract

**Objective:**

To validate the use of a three-dimensional printing system for metric and volumetric analysis of the segments of an abdominal aortic aneurysm (AAA).

**Materials and Methods:**

In patients scheduled to undergo endovascular AAA repair, the computed tomography angiography (CTA) measurements obtained during the preoperative assessment of the patients were compared with those obtained by computed tomography of individualized three-dimensional biomodels.

**Results:**

The volumetric assessment showed a discrepancy of 3-12%, and the difference between the areas was 10-16%.

**Conclusion:**

Computed tomography measurements of 3D-printed biomodels of AAAs appear to be comparable to those of threedimensional CTA measurements of the same AAAs, in terms of the metric and volumetric dimensions.

## Abstract (pt)

**Objetivo:**

Validar a aplicação do método de impressão tridimensional de biomodelos para aferição métrica e volumétrica de segmentos de aneurisma de aorta abdominal.

**Materiais e Métodos:**

Compararam-se as medidas obtidas por tomografia computadorizada dos biomodelos tridimensionais com as realizadas no planejamento pré-operatório de pacientes submetidos a correção endovascular de aneurisma de aorta abdominal.

**Resultados:**

A avaliação da volumetria demonstrou discrepância de 3% a 12% e a diferença entre as áreas foi de 10% a 16%.

**Conclusão:**

A tomografia computadorizada dos biomodelos impressos é compatível nas aferições métricas e volumétricas com as imagens tridimensionais da angiotomografia do paciente.

## INTRODUCTION

An aneurysm is defined as permanent focal dilation of a vessel to at least 50% larger than its normal diameter. The abdominal aorta is the most common site affected, and an abdominal aortic aneurysm (AAA) is characterized by dilation ≥ 3 cm^(1)^. The main risk factors are: male gender, age > 50 years^(2-4)^, smoking^(2-5)^, and having a family history of AAA, especially in first-degree relatives^(2-6)^.

An aneurysm forms as a result of degeneration of the medial layer of an artery, leading to slow, continuous dilation of the vessel lumen^(1)^. Despite the constant production of technical and scientific studies on the formation of aneurysms, there is still no evidence of a direct correlation with atherosclerotic disease, which is typically implicated as the main cause of this process^(7)^. Other causes include trauma, infection, arthritis, cystic necrosis of the tunica media, congenital connective tissue diseases (such as Marfan syndrome), and anastomotic rupture^(1,7)^.

Most patients with AAA are asymptomatic, the diagnosis being made on the basis of incidental findings of imaging examinations performed for other purposes^(1,7)^. The high risk of rupture and potential lethality of an AAA are closely related to the size and rate of growth of the aneurysm^(8)^, surgical treatment being indicated as soon as symptoms arise, the diameter of the aneurysm reaches 5 cm^(1)^, or the aneurysm expands by more than 1 cm in one year^(1)^.

The elective treatment of an AAA can be performed with one of two different techniques^(1)^: conventional open surgery or endovascular repair. Computed tomography angiography (CTA) of the abdominal aorta is used in the surgical planning for both techniques, to determine the type, location, and size of the aneurysm, as well as involvement of the branches of the aorta and the presence of thrombi or calcifications within the aneurysm^(1)^. The tortuosity, angulation, and extent of the intraluminal thrombus should be analyzed, because they influence the risk of periprocedural embolization^(9)^.

In open surgery, the choice of prosthesis is made during the operation, whereas in endovascular treatment it is necessary to measure the segments of the aneurysm in a virtual two-dimensional or three-dimensional (3D) model, with the aim of selecting, prior to surgery, the prosthesis that is best adapted to the morphology of the patient.

The 3D printing process has been gaining ground in the medical field, mainly in preoperative planning, because it produces models analogous to patient anatomy, which facilitates the visualization of structures^(10)^, thus minimizing blood loss during the operation^(11)^, as well as facilitating communication with the patient^(11,12)^. The technique has also been used to create implants, prostheses, and surgical instruments^(11)^.

Because the first 3D printers used only rigid materials, the first surgical specialties to incorporate 3D printing were traumatology and oral/maxillofacial surgery^(11)^. Currently, 3D biomodels are most often used in pelvic and craniofacial surgeries^(13)^, although nearly all surgical specialties have found applications for 3D printing^(11)^.

Endovascular surgery for AAA correction requires detailed preoperative planning in 3D^(13)^, because of the need to assess the branches of the aorta, as well as the proximity to vital organs and structures. When generating a physical model, surgeons have the opportunity to manipulate the anatomical variations of each case and explore the surrounding structures, thus improving preoperative planning^(10)^ and reducing surgical time^(11)^.

Recent studies have shown that preoperative planning using 3D biomodels is more advantageous than is digital 3D imaging. Resident surgery physicians were asked to analyze 3D computer models or 3D printed models and subsequently to formulate a preoperative surgical plan. The group that analyzed the printed models obtained significantly better results in terms of the quality of the surgical plan^(10)^.

The 3D image on the computer presents only information to the visual system of the surgeons, whereas approaching the model with the hands and eyes improves the mental model of the specific anatomy of each patient^(10)^, making it easier for surgeons to plan the steps to be followed during the operation.

The objective of this study was to compare the intraluminal measurements and the volume of AAA segments obtained by computed tomography (CT) of 3D biomodels and by CTA of patients undergoing endovascular correction of an AAA.

## MATERIALS AND METHODS

### Patient selection

From among the medical records of patients hospitalized with characteristics suggestive of an AAA at the University Hospital of Santa Maria, in the city of Santa Maria, Brazil, between January 2016 and November 2017, we selected those of four patients. We selected only patients who had undergone CTA and endovascular treatment.

### 3D reconstruction and creation of the biomodel

**Figure 1:**
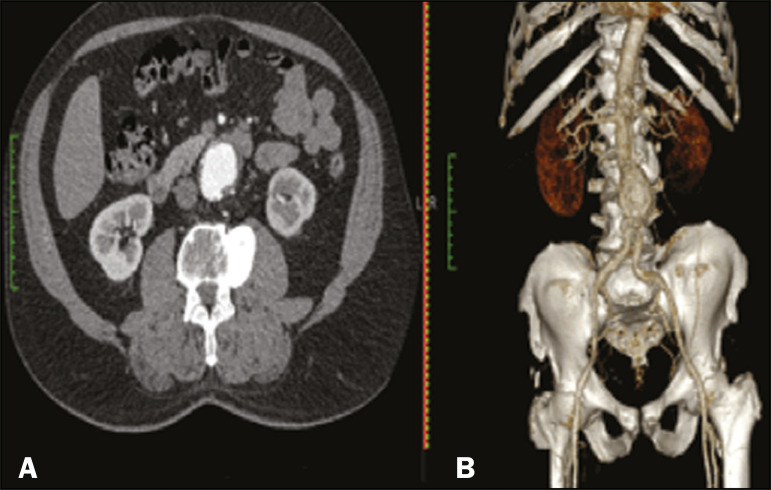
CTA of the abdominal aorta using the Horos software, in axial and 3D views (**A** and **B**, respectively).

**Figure 2:**
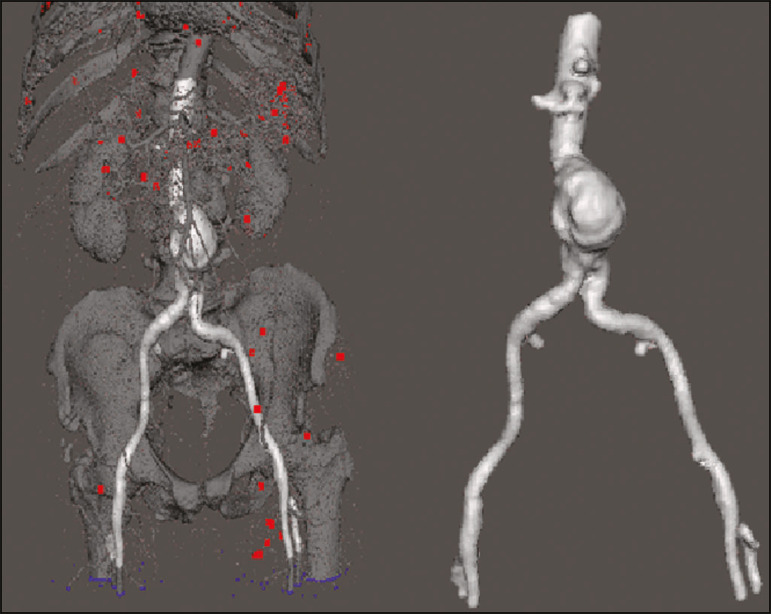
Selection of the region of interest by the Meshmixer software.

The process for creating the 3D biomodels started with the acquisition of sectional CTA images for the selected patients, in the Digital Imaging and Communication in Medicine format of the Horos software, transformed into a 3D file in STL format with the 3DSR tool of the same software (Figure 1). Artifacts surrounding the aneurysm region were excluded using the software Meshmixer, version 2.9.1. (Autodesk, Inc., San Rafael, CA, USA), as depicted in Figure 2. This software imports the Horos data and produces images using scattered point clouds, which are processed in order to obtain dense point clouds, also known as triangular meshes. A point cloud is a set of points expressed in X, Y and Z coordinates and are intended to represent an external surface of an object in 3D.

Eventual filling errors in the mesh were corrected, and the software Netfabb (Autodesk, Inc.) was used in order to segment each model was segmented into four parts, with the objective of saving time and materials. The file was then exported to Cliever Studio 5.1 Pro software (Cliever Tecnologia, Belo Horizonte, Brazil).

The Cliever Studio 5.1 Pro software was developed to manage printouts on machines of the Cliever brand. It is possible to choose the degree of filling, the edge thickness, the distance between layers, and the angle of activation of the ring into which a patient is placed (e.g. gantry)^(14)^. To create parts with an angle < 45° in relation to the print bed, it is necessary to include a support pillar^(15)^.

In the present study, a polylactic acid or polyactide (PLA) was used as a filament. Printing parameters were set as follows: the degree of filling was 5%, the interlayer spacing (layer thickness) was two units, the distance between layers was 0.25 mm, and the angle of activation of the gantry ranged from 30° to 45°, depending on the model of 3D printer employed. These parameters were chosen in order to shorten the printing time and reduce the size of the support pillars, with the objective of saving material and shortening the time to removal of the pillars.

The thickness of the models was increased by 1 mm in order to meet the technical requirements of 3D printing. This condition was necessary to obtain minimum thickness for printing and thus to position the parts of the models on the base, avoiding inaccuracies and losses of material to support the parts.

After a file has been imported, the Cliever Studio 5.1 Pro software automatically provides information such as model height, width, and length, as well as filament size, filament cost, and estimated printing time. For our biomodel, the average printing time was 14 h and the filament cost per biomodel, in Brazilian reals, was approximately R$13.00 (currently equivalent to approximately US$2.50).

**Figure 3:**
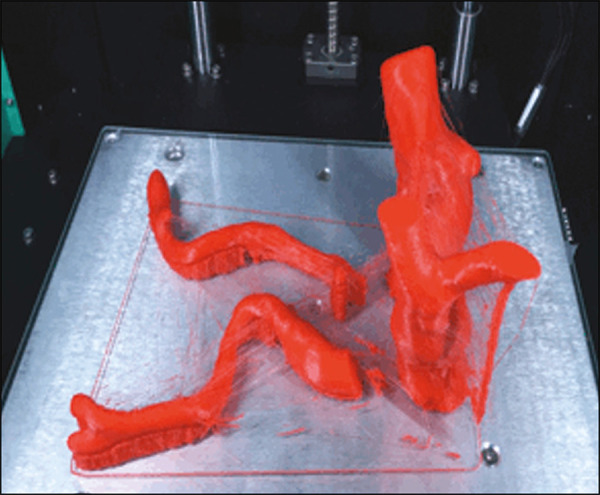
End of the process of printing the biomodel.

**Figure 4:**
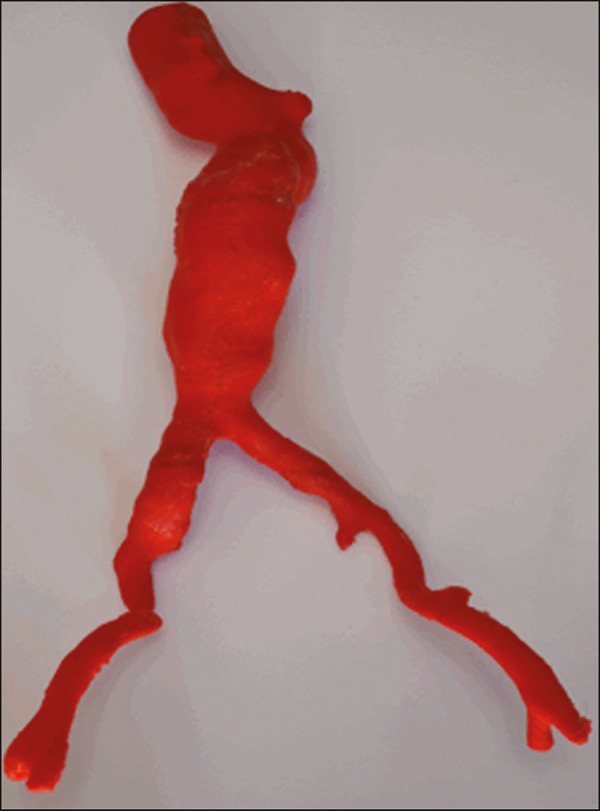
Biomodel after removal of the supports and excess glue.

After the printing process was completed (Figure 3), the support pillars were removed and the four parts of the biomodel were glued together with acetone or cyanoacrylate to form the life-size PLA model of the abdominal aorta for each of the patients selected (Figure 4). Those steps were performed manually.

### Metric and volumetric measurements of the abdominal aortas and of the biomodels

**Figure 5:**
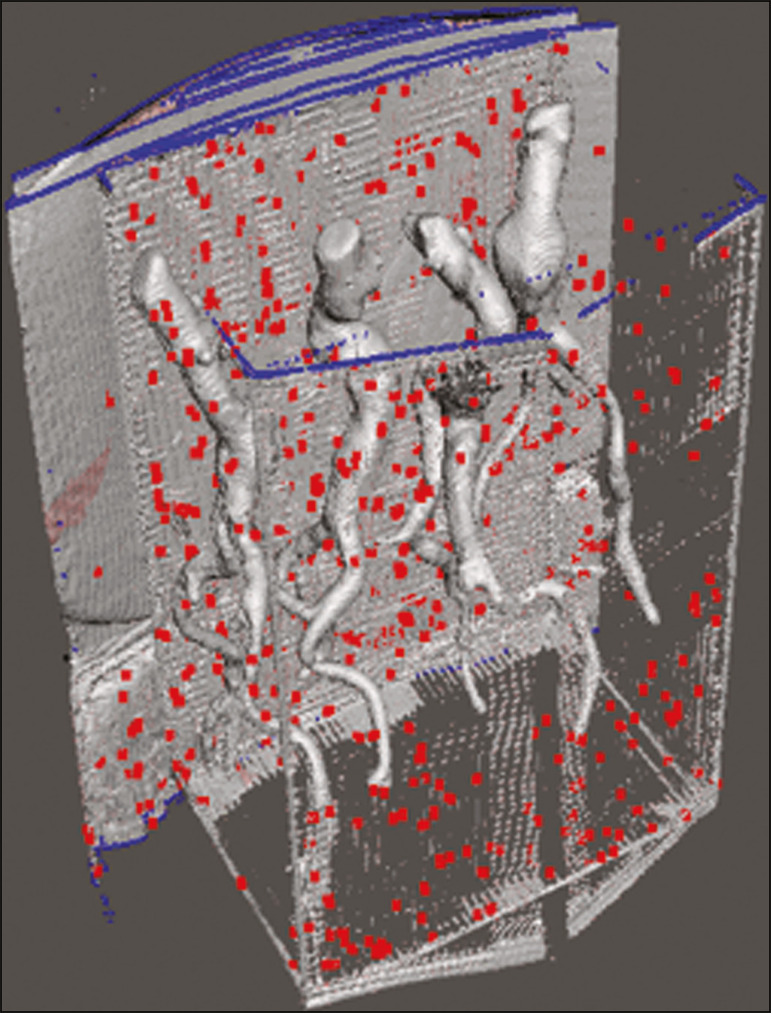
CT before deletion of the image of the cardboard box by the Meshmixer software.

For CT examination, the 4 biomodels were placed inside a cardboard box, separated with Styrofoam (Figure 5). The cardboard box was used in order to reduce costs, allowing us to perform a single CT examination for multiple biomodels, and the Styrofoam dividers were used in order to avoid overlapping of the images. Image artifacts not relevant to the study were eliminated manually using the Meshmixer software.

The measurements of abdominal aortic points on patient CTA examinations were compared with those obtained by CT of the biomodels. Metric measurements were superimposed and compared using the software VRMesh (VirtualGrid, Bellevue, WA, USA), whereas volumetric analyses were performed with the Netfabb software. Because the files came from the same patient, the shared data in each scan had common reference points, so they were automatically aligned.

First, the files referring to the preoperative CTA examinations and CT scans of the 3D-printed biomodels were exported to the VRMesh 3D point cloud, mesh processing software. The models were superimposed and merged into a single model, the color of one of the models being changed to red in order to improve identification by the color tool.

**Figure 6:**
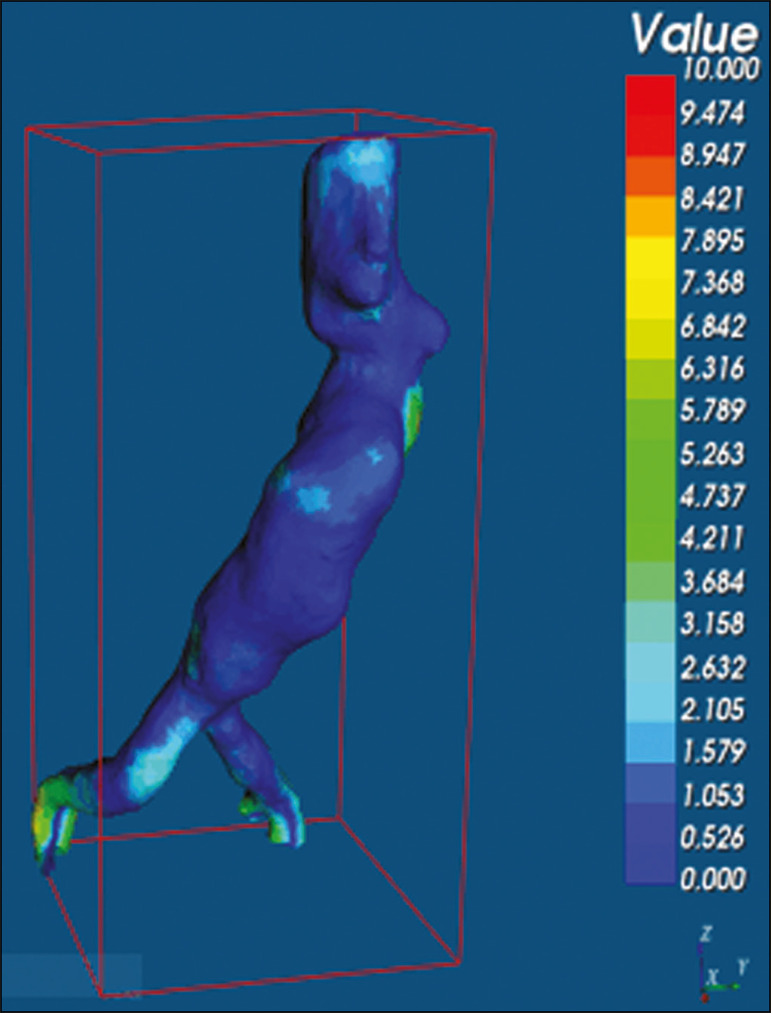
Anterior view of the model and analysis of the differences between the distances, represented in a range of colors, with the VRMesh software.

After the segments of interest had been selected, with the VRMesh editing widget, the branches below the iliac bifurcation were removed. The differences between the files were represented as a range of distances in color, and the acceptable tolerance between the meshes was set at ± 10 mm (Figure 6). The superimposition of the mesh surfaces was checked for accuracy using the VRMesh comparison analysis tools Analyze and Inspection. After the process was completed, the file was exported to Netfabb, which automatically measures volume, area, and height for each model inserted into the system.

## RESULTS

The difference between the 3D CTA model and the 3D-printed biomodel, in terms of the distance between the surfaces of the same segment, was defined within a range of 0-10 mm, calculated in the VRMesh software and represented as a color map in various shades of blue and red. Values above 6.31 mm, shown in red, were found for some points in the distal region and in the glued regions of the biomodel, as shown in Figure 6.

In the four biomodels created, we observed that when the distal ends of the biomodels were excluded, the distances between the 3D CTA model and the 3D-printed biomodel were less than 6.31 mm, as shown in Table 1 and represented in shades of blue in Figure 6. Those measurements did not take into consideration the need for 1-mm increase in the thickness of the models, because of the technical limitations of the 3D printer employed (Cliever). The results of the volumetric analyses of the models in the Netfabb software showed discrepancies of 0-10 mm between the CTA 3D model and the 3D printed biomodel.

**Table 1:** Differences between the CTA 3D model and the 3D-printed biomodel in terms of the surface measurements calculated by the VRMesh software.

Patient	CTA volume (mm^3^)	PLA volume (mm^3^)	PLA/CTA volume ratio	CTA area (mm^2^)	PLA area (mm^2^)	PLA/CTA area ratio
A	164.89	184.92	1.12	327.10	377.11	1.15
B	197.09	202.48	1.03	366.12	420.56	1.15
C	179.90	199.56	1.11	432.37	474.47	1.10
D	213.19	220.11	1.03	442.49	514.63	1.16

## DISCUSSION

The past decade has seen a remarkable growth in the use of 3D printing in medicine. The growth was driven by the development of high-resolution image studies, together with the rapid development of 3D-printing techniques and the development of new printing materials. These advances have resulted in cost reductions associated with the creation of high-resolution medical biomodels. The evolution of this disruptive technology has revolutionized medical practice.

In certain scenarios, it can be difficult to align the original (preoperative CTA) STL model with the result of the CT scan of the 3D-printed model. Although there are automatic alignment tools in some software, such as VRMesh, the accuracy of the alignment must be carefully evaluated, and manual adjustment may be necessary. Misalignment will result in errors in the qualitative assessment process. In addition, the appropriate imaging modality and imaging protocols must be used. This method also presents challenges for models built with flexible materials. Because such models may become deformed after being printed, there is no guarantee that the model will retain its original shape during scanning. Therefore, it is useful to construct structures to support the model during its construction, so that it retains the shape originally observed in the patient.

Biomodels now have various applications in medical practice. Therefore, it is expected that the replicas will have metric and volumetric measurements that are reliable representations of the anatomy of the patient. The use of a biomodel is important mainly in diseases in which interindividual anatomical variations are relevant, such as aortic aneurysm. Images obtained with CTA allow virtual 3D visualization of the disease in question. However, with a printed biomodel, the surgeon can perform preoperative planning that is more comprehensive, analyzing the details in a visual and tactile way, and can handle the model in order to facilitate the simulation in complex cases. Recent studies have demonstrated the various benefits of using a 3D-printed model, including better preoperative planning, better patient understanding of the procedure, and shorter surgical times^(10,11)^.

The use of different imaging methods can result in differences in the measured values-for example, the diameter of an aneurysm is typically 2 mm larger when measured on CT than when measured by ultrasound^(16)^. It should be noted that in the present study, the use of biomodels to measure the AAA segment produced similar results, except at the distal end. That might be due to errors in the manual removal of the support pillars, manual gluing, or the fitting when the final model was being constructed. That type of discrepancy, observed in some of the biomodels, would have no influence on the preoperative planning of AAA, and can therefore be discounted, because it was located below the iliac bifurcation. Despite the small number of patients included in this study and the manual steps in the process, the differences in area and volume obtained with the Netfabb software did not exceed 12% and 16%, respectively.

In the literature, an intra-observer or inter-observer variation in the CT measurement of aortic aneurysm diameter of 2-5 mm is considered to be within the normal range, whereas values > 5 mm are considered significant^(17)^. To our knowledge, there have been no studies aimed at determining the minimum, mean, and maximum discrepancy between a 3D-printed biomodel and the anatomy of the patient.

## CONCLUSION

Recent technological innovations applied in healthcare, such as minimally invasive techniques that are associated with greater patient well-being, are defining the road ahead. Therefore, it is necessary to outline the strengths and weaknesses of 3D printing.

In the present study, it was concluded that, although the biomodels were consistent with the 3D CTA images of the patients, in terms of the metric and volumetric measurements, such models cannot be used in the preoperative planning of abdominal aortic aneurysmectomy, a procedure that requires the measurement of mural thrombi.

One of the main scientific challenges of the additive manufacturing technique for the representation of an aortic aneurysm is to make it feasible to print the mural thrombi, separating them from adjacent organs of the same density on the CT gray scale. Therefore, there is a need for further studies, involving larger samples, to determine whether the biomodel represents a true replica of the human anatomy and can be used as surgical planning, as well as to educate physicians and residents in training.
